# Metabolic Patterning on a Chip: Towards *in vitro* Liver Zonation of Primary Rat and Human Hepatocytes

**DOI:** 10.1038/s41598-018-27179-6

**Published:** 2018-06-12

**Authors:** Young Bok (Abraham) Kang, Jinsu Eo, Safak Mert, Martin L. Yarmush, O. Berk Usta

**Affiliations:** 1 Center for Engineering in Medicine, Department of Surgery, Massachusetts General Hospital, Harvard Medical School, and Shriners Hospitals for Children-Boston, Boston, MA USA; 2 Department of Biomedical Engineering, Rutgers University, 599 Taylor Rd., Piscataway, NJ USA

## Abstract

An important number of healthy and diseased tissues shows spatial variations in their metabolic capacities across the tissue. The liver is a prime example of such heterogeneity where the gradual changes in various metabolic activities across the liver sinusoid is termed as “zonation” of the liver. Here, we introduce the Metabolic Patterning on a Chip (MPOC) platform capable of dynamically creating metabolic patterns across the length of a microchamber of liver tissue via actively enforced gradients of various metabolic modulators such as hormones and inducers. Using this platform, we were able to create continuous liver tissues of both rat and human origin with gradually changing metabolic activities. The gradients we have created in nitrogen, carbohydrate and xenobiotic metabolisms recapitulated an *in vivo* like zonation and zonal toxic response. Beyond its application in recapitulation of liver zonation *in vitro* as we demonstrate here, the MPOC platform can be used and expanded for a variety of purposes including better understanding of heterogeneity in many different tissues during developmental and adult stages.

## Introduction

Many organs and tissues display spatial heterogeneity along their depth or their primary axis of blood flow. This heterogeneity manifests itself in terms of morphology, gene expression regulating different functions, and most importantly as metabolic activities both in health and disease. Rather than only differences in the cellular composition (i.e. ratios of different cell types), these metabolic patterns can also be observed amongst the same cell type in different locations. Such variability can stem from either signaling events during development^1,2^ which result in stable gene expression and metabolic patterns in the tissue; or from dynamic gradients of modulators such as nutrients, oxygen, cytokines and other signaling molecules which result in a more dynamic pattern of tissue behavior. Heterogeneity in metabolic and gene makeup in a tissue can, in turn, give rise to spatially variable toxicity^3^, efficacy, and disease^4,5^ response. For example, as tumors grow, they display strong patterns in their phenotype due to oxygen gradients^6^ and Wnt signaling activators and inhibitors^7^, to the extent that these patterns are responsible for their resistance to chemo/radio therapies^8^. Recent studies demonstrate that pancreatic islets also show two distinct subpopulations where the dorsal pancreas gives rise to islets with a higher proportion of somatostatin and glucagon expressing cells, while the ventral pancreas contains islets rich in pancreatic polypeptide cells^9,10^.

**Figure 1:**
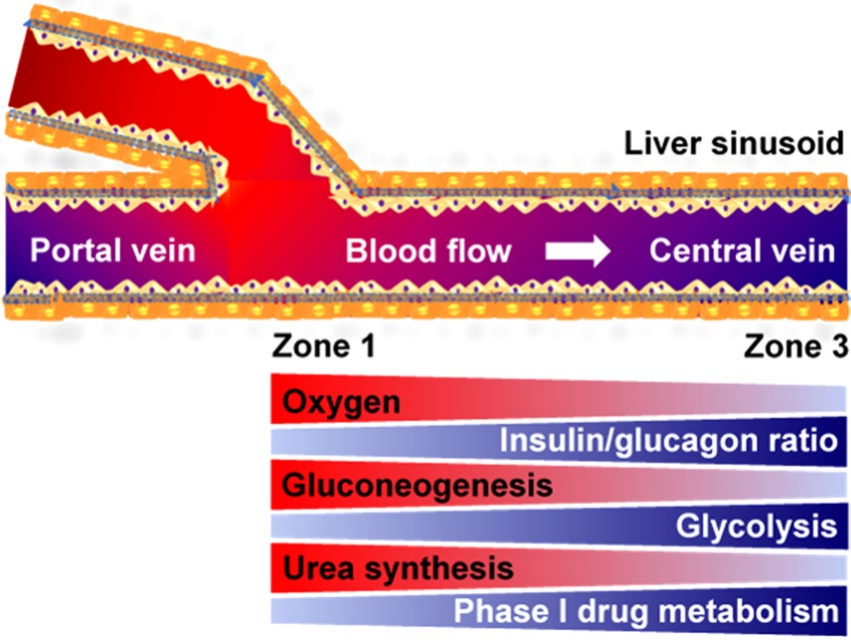
Liver zonation overview. Hepatocytes, which play a predominant role in various metabolisms in the liver, show a remarkable heterogeneity across the liver sinusoid in terms of morphology and metabolism known as liver metabolic zonation. Hepatocytes in Zone 1 where oxygen-rich blood from hepatic artery blends with nutrient-rich blood from portal vein have high activities in oxygen-related metabolism, gluconeogenesis, urea synthesis, and ammonia detoxification. In contrast, hepatocytes in Zone 3 where the concentration of oxygen and hormone ratio (glucagon to insulin ratio) are low around the central vein, have high activities in glycolysis and phase 1 drug metabolism.

The liver is, perhaps, the most prolific tissue in terms of its cellular heterogeneity where the gradual changes in various functional expression and metabolic activities across the liver sinusoid have a specific name: “zonation” of the liver (Fig. 1). The metabolic gradients in the liver occur from periportal (Zone 1, PP) region of the liver sinusoid to the perivenous (Zone 3, PV) region^11,12^. Gradients (Zone 1 to Zone 3) in function have been observed, especially in hepatocytes, as heterogeneity of morphology; nitrogen, carbohydrate, xenobiotic, and others metabolisms along with critical functional capacities such as protein synthesis. While some of this heterogeneity stem from micro-environmental gradients in hormonal, nutrient and oxygen gradients^13–15^ across the sinusoid to create “dynamic” functional gradients, recent studies^1,16^ highlight the importance of Wnt/β-Catenin signaling pathway towards creating “static” zonal differences. In general, Zone 1 hepatocytes (parenchymal cells of the liver) are efficient at oxidative metabolism, fatty acid oxidation, gluconeogenesis, bile acid extraction, ammonia detoxification, and urea and glutathione conjugation. Zone 3 hepatocytes are efficient at glycolysis, liponeogenesis, and CYP450 biotransformation^17–20^. Of special significance for its role in understanding drug toxicity, the xenobiotic (drug) metabolism predominantly occurs in Zone 3 hepatocytes, which is especially true for Phase I metabolic activity. In contrast, elevated levels of some Phase II detoxification activities can also be observed in Zone 1.

In general, these naturally occurring metabolic and phenotypic patterns, in the liver along with other tissues of importance, are rarely if ever recapitulated in *in vitro* systems; yet they might very well be the key to understand many unexpected and critical behavior in health and disease. While there have been a few attempts^15,21–24^ to create a zonated liver in a microfluidic format, these efforts have mostly been limited to the creation of oxygen gradients in a passive manner, ignoring other modulators of metabolism such as hormones. A more recent study^23^ towards liver zonation created two *“separate”* micro-devices for Zone 1 and Zone 3 each with defined oxygen inputs and passively monitoring the oxygen gradients. The use of separate micro-devices hampers the scalability and thus the throughput of this approach, and makes it challenging to understand the gradual change in dynamics from one zone to the other. An easy to use *in vitro* preclinical model, which presents a connected tissue, as *in vivo*, with the ability to create gradual and/or arbitrary metabolic patterns, and in a physiologically relevant microfluidic format would be of tremendous use for studying biological heterogeneity.

Here, we introduce the concept of “microfluidic metabolic patterning” which takes advantage of advances in the microfluidic tissue culture realm towards spatial control of the microenvironment and enforcing a desired spatial pattern of the metabolic activity across the microenvironment. To this end, we have designed a new microfluidic platform, dubbed the “Metabolic Patterning on a Chip (MPOC) device”, which consists of a microfluidic gradient generator and a microfluidic tissue culture chamber with two orthogonal axes for patterning flows; and cell seeding and/or secondary flows if necessary. This new platform stemmed from our previous work where we aimed to mimic liver zonation in a narrow prototype microfluidic chip^13^. While this proof-of-concept work allowed us to demonstrate the possibility of creating zone like responses in a microfluidic format, it had several drawbacks. These included, among others, the difficulty of sectional sampling for fluids as well as the cells for spatial characterization of the patterned tissue responses. While microfluidic gradient generators have been used previously in biological context especially for migration assays^25,26^, their use in whole tissue culture as we describe here is uncommon. The MPOC platform is capable of generating a differential concentration pattern of different metabolic modulators such as hormones, enzymatic inducers, and other small molecules across the width of the cell culture chamber to induce a desired metabolic pattern across the cultured tissue. Moreover, this new platform now allows us to sample and assay in a sectional manner as necessary.

In this work, we demonstrate the applicability of the MPOC platform by using it towards recapitulating several aspects of liver zonation *in vitro* since liver displays one of the richest and most prominent metabolic patterning among different tissues of interest. The MPOC device provides a single continuous tissue and the possibility to look at a wide range of modulators individually and/or in combination with one another in a systematic manner, unlike some other models. Additionally, in contrast with methods that rely on passive cellular consumption for a particular modulator, the MPOC device predefines and enforces the concentration of all the modulators, and thus the metabolic pattern, to allow for a precise scientific study and hypothesis testing that goes beyond just recapitulating *in vivo* phenomena. While cellular consumption is partially responsible for the concentration and in turn metabolic gradients observed *in vivo*, relying on them in *in vitro* poses an important risk due to the high variability of cellular metabolism in different donors especially for human subjects. Moreover, each metabolism is altered differently in an *in vitro* system compared to their *in vivo* situation, thus optimization of the device for proper patterning, i.e. recapitulation, of each metabolism becomes a nearly impossible task if one relies simply on passive cellular consumption of the input. While not demonstrated in this work, the MPOC device is capable of a secondary flow that is orthogonal to the patterning flow once the active patterning is finished; this allows for switching to the passive consumption based gradient formation where appropriate. We, thus, consider the concept of metabolic patterning and MPOC platform to be versatile tools with many applications, which can be further refined and expanded to study a plethora of different tissues and test many hypotheses both in healthy and diseased tissues, in a finely tuned manner.

In what follows, we first describe the design and operation of the MPOC platform, and methods towards its use for recreating liver zonation for primary rat and human hepatocytes *in vitro* in *section 2 (Materials and Methods)*. We then demonstrate the metabolic patterning of the liver tissue in the MPOC device for carbohydrate/glucose, nitrogen, and xenobiotic metabolisms and a zone-like toxic response of the liver tissues in *section 3 (Results and Discussion)*. In this study, we only present patterning of the different metabolisms by one modulator (hormones and enzymatic inducers) at a time. We then give a brief conclusion and outlook about the use of MPOC platform in the future towards studying other patterned *in vivo* phenomena and its repurposing for other questions in *section 4 (Conclusions and Outlook)*.

## Materials and Methods

### Microfluidic device design and fabrication

**Figure 2:**
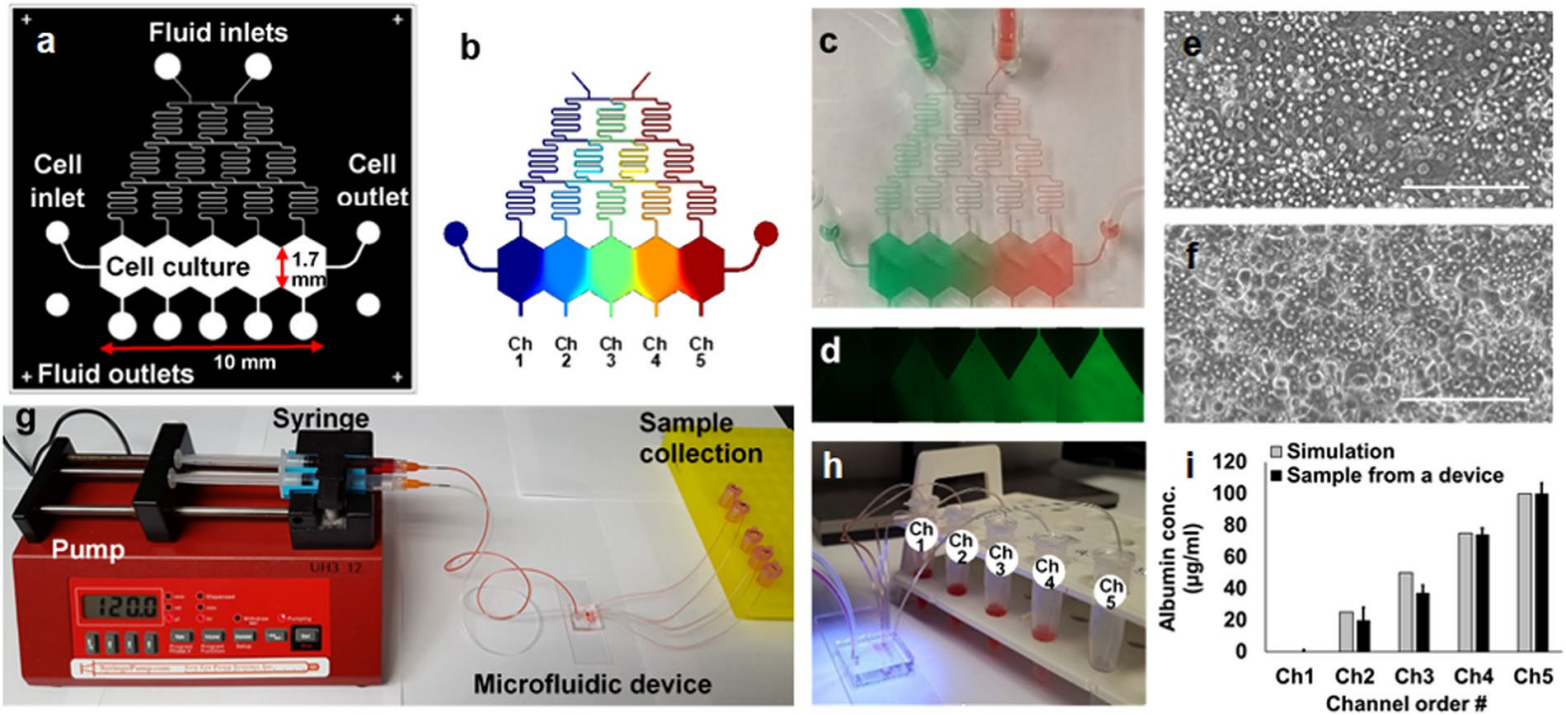
The “Metabolic Patterning on a Chip” (MPOC) platform. (**a**) Drawing of a microfluidic device with a gradient generator. (**b**) A gradient of concentration simulated by COMSOL^®^ Multiphysics. A gradient of (**c**) food dye and (**d**) fluorescein in the microfluidic device. Cell morphology of primary (**e**) rat and (**f**) human hepatocytes in the microfluidic device at day 1. Scale bar: 200 µm. (**g**) Continuous perfusion system with a syringe pump, a microfluidic device, and sample collection. (**h**) A sample collection from the device. (**i**) We collected media samples from each outlet of five channels, which was connected to a 1.5 ml collection tube via a Tygon tubing, after generating a gradient of bovine serum albumin (BSA, one inlet: 0 µg/mL BSA in PBS and the other inlet: 100 µg/mL BSA in PBS) under cell-free condition. BSA concentration levels of samples were measured as approximately 0, 20, 37, 74, and 100 µg/mL, respectively, using Pierce BCA protein assay kit (23227, Thermofisher Scientific) by spectrophotometer (Biorad) (ANOVA n = 3, p < 0.05). These outcomes were similar to those simulated by COMSOL^®^ Multiphysics.

Our microfluidic MPOC device consists of media fluid inlets/outlets, a cell seeding inlet/outlet, a gradient generator, and a cell culture chamber as depicted in Fig. 2. The gradient generator has a typical Christmas-tree structure (width (w) × height (h): 75 × 200 µm) to create a gradient of concentrations between the two extreme input values at the inlets. The tree structure is terminated at five branches connected to five sections, with an elongated hexagon shape, of the cell culture chamber (wiggle shape, w × h × length (l): 10,000 × 200 × 1,700 µm). Five outlets are connected to these sections to enable sectional sample collection (Fig. 2g–i). We have designed two versions of the cell culture chamber; (a) a continuous cell culture chamber without any barriers between the culture areas was designed to allow cell-cell interaction between each section and was used for cell image analysis (Fig. 2a), (b) we placed physical barriers (w × h: 100 × 200 µm) between each section to enable easier sectional cell collection (Fig. S1).

PDMS replica molding was used based on an SU-8 template on a silicon wafer manufactured via photolithography technology. Inlets for perfusion and cellular seeding, and outlet ports for sectional sample collection were punched into the peeled-off PDMS using a biopsy punch (Miltex Inc, Plainsboro, NJ, USA) of 1.5 mm, 0.75 mm, and 0.75 mm diameters, respectively. The PDMS molds were bonded on glass microscope slides (75 × 25 mm, Thermofisher Scientific, Grand Island, NY, USA) after oxygen plasma treatment by cross-aligning five outlets of the cell culture chamber to the side edge line of the glass microscope slide in order to flow out of the chamber with less pressure resistance.

### Hepatocyte isolation and culture

Adult female Lewis rats (Charles River Laboratories, MA) were used for isolation of primary rat hepatocytes as described previously^27,28^. This isolation was performed by the Cell Resource Core (CRC) according to the protocol #2011N000111 approved by the Institutional Animal Care and Use Committee (IACUC) at the Massachusetts General Hospital (MGH). Approximately 3.1–4.1 × 10^4^ cells, with 90–95% viability, were plated in the microfluidic device coated with 50 µg/mL fibronectin (Sigma-Aldrich, St. Louis, MO, USA) for 20–40 min at 37 °C. The cells were washed with fresh media within 1 hour after plating cells. Dulbecco’s modified eagle’s medium (DMEM, Life Technologies, Carlsbad, CA, USA) supplemented with 10% fetal bovine serum (FBS, Sigma, St. Louis, MO, USA), 0.5 U/mL insulin, 7 ng/mL glucagon, 20  ng/mL epidermal growth factor, 7.5 μg/mL hydrocortisone, 200 U/mL penicillin, 200 μg/mL streptomycin, and 50 μg/mL gentamycin was used for culturing primary rat hepatocytes.

Cryopreserved primary human hepatocytes were obtained from either the Triangle Research Laboratory (TRL, Durham, NC, USA) or the CRC at the MGH (Boston, MA, USA). The CRC conducts all primary human hepatocyte isolation procedures according to the protocol #2012P001090 approved by the IACUC at the MGH. The cells (Lot no. 4119E, TRL and Lot no. HW 54–1, CRC) were thawed in human hepatocyte thawing medium (MCHT50, TRL, Durham, NC, USA) according to the manufacturer’s protocol. The 5.9–6.8 × 10^4^ cells with 80–90% viability after thawing the cells were plated in the microfluidic device that was coated with fibronectin (Sigma-Aldrich) of 50 µg/mL for 20–40 min at 37 °C, followed by a rat tail collagen type I coating of 50 µg/mL for 20–40 min at 37 °C. The cells were washed with fresh plating media (MP100, TRL) 1 hour after plating cells. The media was replaced with basal, serum-free William’s E medium (WEB media) supplemented with 2 mM glutamine, 200 U/mL penicillin, and 200 μg/mL streptomycin 24 hours after cell seeding for cell induction. The primary rat/human hepatocytes were incubated at 37 °C in 10% CO_2_.

### Induction of cells with chemicals or hormones

For microfluidic perfusion culture of hepatocytes, fluid inlets of the device were connected to syringes loaded with the culture medium (with or without inducers) via a Tygon tubing. Cell seeding inlet and outlet were blocked by putting a tied Tygon tubing into the ports. Cells were induced by a gradient of inducers generated by mixing WEB media supplemented with 100 nM glucagon and WEB media with 100 U/L insulin for the patterning of carbohydrate/nitrogen metabolism or by mixing WEB media and WEB media supplemented with 2 µM 3-methylcholanthrene (3-MC) for creating the patterning of carbohydrate/nitrogen metabolism for the patterning of xenobiotic metabolism at a flow rate of 30–120 µL/hr (Fig. 2).

### Periodic acid-schiff (PAS) staining

After induction of hepatocytes with a hormone ratio (insulin/glucagon) gradient for the prescribed interval (24 hours), the microfluidic device was disconnected from the syringe pumps. Cells were, then, fixed with 100% methanol for 20 min at −20 °C. The PDMS microfluidic device was peeled off from the glass microscope slide by cutting around the pattern of cell culture chamber of PDMS layer on the surface of glass microscope slide with a razor blade before further analysis. Cells were treated with PAS reagent (Sigma-Aldrich, St. Louis, MO, USA) according to the manufacturer’s instruction to assess the level of glycogen in the primary hepatocytes as a marker of carbohydrate metabolism in hepatocytes. Images were captured on a Nikon microscope (Diaphot TMD, Nikon, Tokyo, Japan) at a 10X magnification with an attached Nikon D5100 digital SLR camera.

### Immunofluorescence assay

For immunofluorescence assays, the cellular induction was terminated after the prescribed interval (24 hours) by turning off and disconnecting the syringe pumps. Cells were fixed with 100% methanol for 20 min at −20 °C or 4% paraformaldehyde for 15 min at room temperature. The PDMS microfluidic devices were detached from the glass microscope slides by cutting around the pattern of cell culture chamber of PDMS layer on the surface of glass microscope slide with a razor blade for further analysis. Cells, still attached to the glass slides, were then incubated with phosphate-buffered saline (PBS) containing 0.25–0.5% Triton X-100 for 10 min to improve the penetration of antibodies. Cells were then incubated with 1% bovine serum albumin (BSA) and 22.52 mg/ml glycine in PBST (PBS + 0.1% Tween 20) for 30 min to block non-specific antibody binding. Cells were incubated overnight at 4 °C with primary antibodies in 1% BSA in PBST: carbamoyl phosphate synthetase 1 (a marker for urea production by the ornithine cycle, CPS1, 1:100, ab3682/ab45956, Abcam, Cambridge, MA, USA) or cytochrome P450 1A1/1A2 (CYP1A1/1A2, 1:100, ab22717, Abcam). Cells were incubated with the following secondary antibodies in 1% BSA for 1 hour at room temperature in the dark: Alexa Fluor 488 (1:1000, ab150073, Abcam or R10477, Thermofisher Scientific) and goat anti-mouse conjugated secondary antibody (Texas Red, 1:1000, ab6787, Abcam). After incubation of the secondary antibodies, the cells were washed 3 times with PBS. Nuclei were counterstained with Hoechst (1:2000, 33258, Thermofisher Scientific) and images were captured on an EVOS fluorescence microscope.

### CYP activity-dependent response to acetaminophen in hepatocytes

To estimate the drug-induced liver toxicity via CYP activity-dependent response to acetaminophen, cells were induced with a gradient of 2 µM 3-MC in the device for 24 hours. After induction, cells were exposed to 10 mM acetaminophen, a sublethal dose^29^, in WEB media for 4 hours at 37 °C. Primary hepatocytes were stained with tetramethylrhodamine methyl ester (TMRM; 500 nM in WEB media) for about 30 min at 37 °C before and after acetaminophen dose, to monitor the mitochondrial membrane potential as an indicator of cell viability. All images were captured on an EVOS fluorescence microscope (EVOS FL, Thermofisher Scientific) at a 10X magnification.

### RNA isolation and RT-PCR analysis

After induction of hepatocytes with the inducer (3-MC), hepatocytes were collected from each section of the device with barriers. Total RNA was extracted from hepatocytes with Trizol reagent (Thermofisher Scientific) according to the manufacturer’s instruction. Total RNA (10–100 ng) was reverse-transcribed into cDNA using cDNA synthesis kit (iScript, Bio-rad, Portland, ME, USA) following the manufacturer’s instruction. Real-time quantitative PCR was performed by using the product of cDNA synthesis and power SYBR Green PCR master mix kit (Life Technologies, Carlsbad, CA, USA) in ViiA 7 Real-time PCR system (Life Technologies) according to the manufacturer’s instructions. The following PCR primer pairs for human genes were used: CYP1A2, 5-CTTCGGACAGCACTTCCCTG-3 and 5-AGGGTTAGGCAGGTAGCGAA-3 (133 bp); β-actin, 5-CCTCGCCTTTGCCGATCC-3 and 5- GCGCGGCGATATCATCATCC-3 (71 bp). The thermal cycling conditions were as follows; 50 °C for 2 min, 95 °C for 10 min, 42 cycles at 95 °C for 14 s and 60 °C for 1 min. The mRNA expression level of all target genes was analyzed in duplicate by comparative Ct (ΔΔCt) method and normalized to the mRNA expression level of β-actin.

### Image quantification

All microscopic images obtained from PAS and fluorescent staining assays were converted to greyscale images for image quantification. The average of greyscale/fluorescence intensity of the stained cells across the width of each device was quantified via built-in ImageJ functions (ImageJ, NIH, Bethesda, MD, USA). The measured average intensity of each image was re-scaled to percentage scale by normalizing it with the minimum and maximum intensity of (non-)induced cell images in a device. All data were plotted by curve fitting with non-linear regression.

### Statistical analysis

The quantitative data are presented as the mean ± standard error of the mean (SEM) from three to seven devices (n = 3–7). The fixed effect of induction (induced vs. non-induced) and the repeated effect of location within the device (% device width) on cell viability or staining intensity was assessed using one-way ANOVA and Tukey’s test. P-values less than 0.05 were considered statistically significant.

### Data availability

All data generated or analyzed during this study are included in this published article.

## Results and Discussion

### Characterization of the microfluidic device with the gradient generator

**Figure 3:**
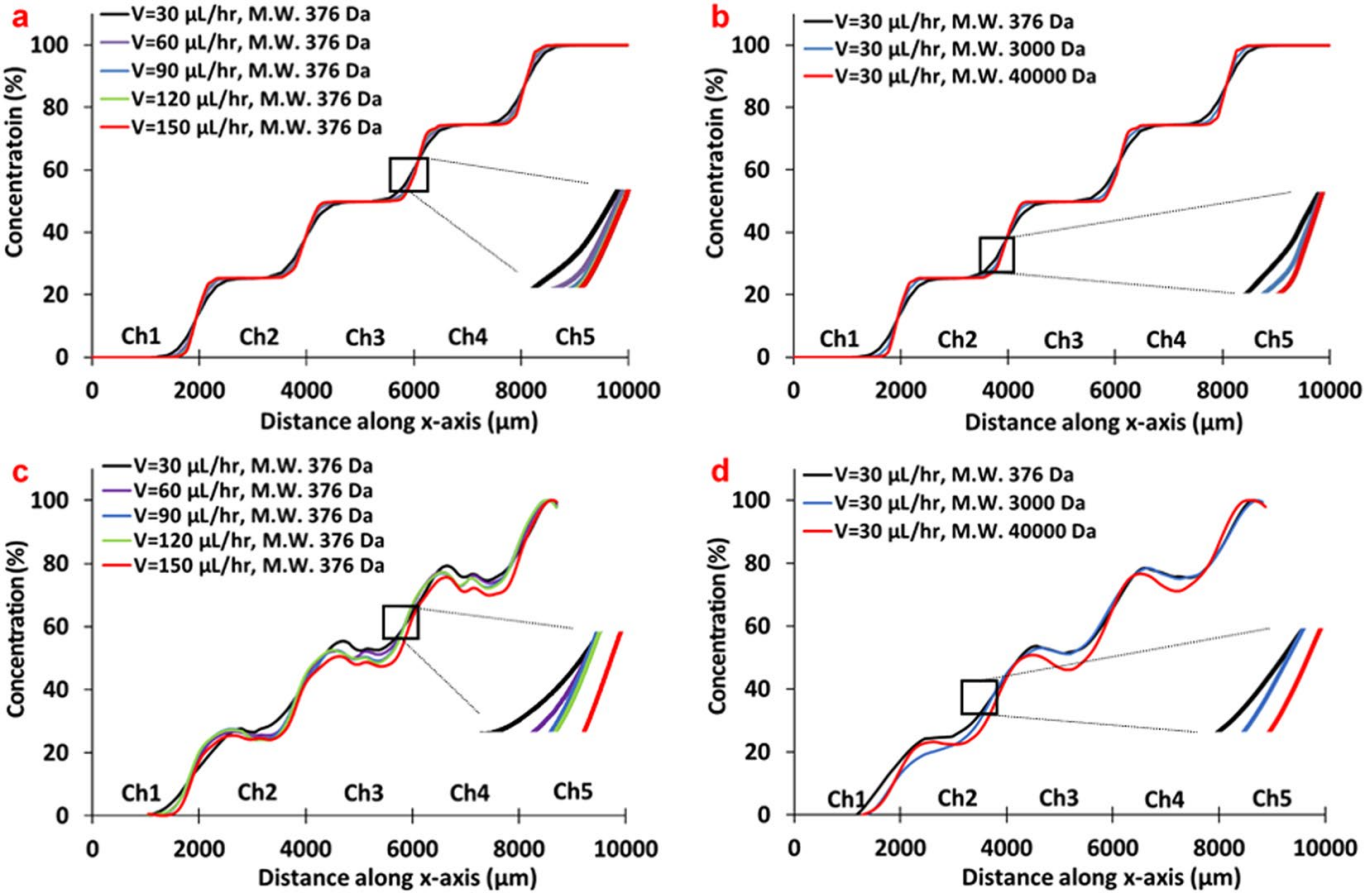
The characterization of gradient generation in the MPOC platform. The effect of (**a**) flow rate and (**b**) diffusion coefficients at a flow rate of 30 µL/hr on a gradient profile by COMSOL^®^ Multiphysics. The effect of (**c**) a flow rate and (**d**) diffusion coefficients at a flow rate of 30 µL/hr on a gradient profile by an experiment using fluorescently-labeled solution with different molecular weight (n = 3, standard error of the mean (SEM) <±7.0).

In order to establish the flow and dispersion characteristics in our MPOC device we have conducted both computational and laboratory experiments at different flow rates and various model diffusing species. The concentration profiles, across the width of the cell culture chamber area of the MPOC device, at the midpoint of channels (Fig. 2a,b) were compared from the computational and laboratory experiments. We first simulated the effect of flow rate and diffusion coefficient of suspended species on the gradient profile in the microfluidic device using COMSOL^®^ Multiphysics (COMSOL Inc. Burlington, MA, USA). The microfluidic device geometry, drawn by CAD software, was imported to the COMSOL^®^ Multiphysics. Flow rate range of 30–150 µL/hr per each inlet of the gradient generator was used to investigate the effect of the flow rate on the concentration profile at a fixed molecular weight of 376 Da corresponding, roughly, to the diffusion coefficient of 3-MC and that of fluorescein. As the volumetric flow rate increased from 30 µL/hr to 150 µL/hr, the rate of dispersion between channels decreased as expected (Fig. 3a). An almost stepwise ascending gradient profile of concentration was observed along the width of the cell culture chamber at the highest flow rate. To demonstrate the effect of the diffusion coefficient on the concentration profile, we used a diffusion coefficient range of 4.25 × 10^−10^–6.4 × 10^−11^ m^2^s^−1^ to reflect the diffusivity of species (3-MC, glucagon, and insulin) used in our cell culture experiments (Table 1) and a fixed flow rate of 30 µL/hr. As the diffusivity increased (lower molecular weight), the concentration profile changed from a strict stepwise profile with constant concentration across the whole channel width to slightly more gradual profiles with higher dispersion between the adjacent sections (Fig. 3b).

**Table 1:** The molecular weight and diffusion coefficient of materials.

Material	Molecular weight (Da)	Diffusion coefficient (m^2^s^−1^)	Ref.
Fluorescein (F6377, Sigma-Aldrich)	376	4.25 ± 0.1 × 10^−10^ (in water)	^58^
Fluorescein-isothiocyanate (FITC)-dextran (D3305, Thermofisher scientific)	3,000	1.79 × 10^−10^	^59^
Texas red-dextran(D1829, Thermofisher scientific)	40,000	6.4 ± 0.02 × 10^−11^	^60^
3-Methylcholanthrene (Sigma-Aldrich)	268.36	8.0 × 10^−10^ (in water)	^61^
Glucagon	3,482.75	—	^62^
Insulin	5,807.57	1.5 × 10^−10^ (in water)	^62^

Next, we conducted laboratory experiments and verified both the effect of the volumetric flow rate and the molecular weight of inducers on the gradient profile. As a proof of concept, we initially observed the formation of a gradient pattern in our microfluidic device using food colorings with a flow rate of 30 µL/hr per each inlet of the gradient generator (Fig. 2c). After that, we sought to replicate the computer simulations by using three fluorescently-labeled particles with various molecular weights; fluorescein (376 Da, F6377, Sigma-Aldrich), fluorescein isothiocyanate (FITC)-dextran (3 kDa, D3305, Thermofisher Scientific), and Texas Red-dextran (40 kDa, D1829, Thermofisher Scientific) (Table 1). These molecular weights roughly correspond to the diffusivity of 3-MC, glucagon, insulin, and other larger proteins, which we use in our cell culture experiments. A 2 mM solution of the fluorescently labeled reagent in PBS was introduced to one of the inlets of the gradient generator and pure PBS in the other inlet with volumetric flow rates of 30, 60, 90, 120 and 150 µL/hr. After the flow pattern was stabilized, fluorescence images were obtained from the device using the EVOS fluorescence microscope (Fig. 2d). The fluorescence intensity of images was analyzed using Image J to estimate their concentration in the device. The experimental results (Fig. 3c,d) closely resembled the computational studies albeit with few imaging artifacts; the concentration dips in the midpoint of each separate section is likely due to the stitching of images. Together, these computational and experimental results show that our device is capable of generating stable concentration gradients of species that fall in the molecular weight range of reagents (inducers, hormones, etc.) we are interested in using in our cell culture experiments.

Hepatocytes, in their natural environment, do not experience any shear stress since they are shielded by the endothelium and the Space of Disse, which separates them from direct exposure to blood flow. Nevertheless, several reports indicate that they can not only withstand low shear stresses but such stresses can help stabilize their function *in vitro*^30–34^. Accordingly, we estimated the magnitude of the shear stress exerted in our device under the flow rates of interest to avoid adversely affecting the hepatocytes’ health. Our estimation is based on a total inlet volumetric flow rate range of 60–300 µL/hr and the dimensions of each sectional channel (w × h: 2,000 × 200 µm) in the cell culture chamber. With the assumption of a steady-state laminar flow profile in the device, we calculated the maximum shear stress at the bottom of the cell culture chamber to be approximately 0.0025–0.013 dynes/cm^2^ according to the following equation (1):

1 $${\rm{\tau }}=\frac{6{\rm{\mu }}Q}{{{\rm{h}}}^{2}{\rm{w}}}$$

where µ = flow viscosity (kg/m*s), Q = volumetric flow rate (m^3^/s), h = channel height (m), and w = channel width (m)^35^. We have also analyzed the flow profile and shear stress with computational studies with the same parameter ranges (Fig. S2) and, in line with our calculations, we found that our microfluidic device provides a perfusion system with an acceptably low shear-stress environment^36^.

### Zonation of carbohydrate and glucose metabolism in hepatocytes

The liver plays a critical role in maintaining the balance of glycogen - the main carbohydrate stored in the liver - and glucose, and their homeostasis. Insulin and glucagon are the two regulatory hormones that are critical to this homeostasis and along with Wnt/β-catenin signaling they regulate many aspects of the carbohydrate/glucose metabolism^37,38^. In normal physiology, a glucagon/insulin gradient exists along the liver sinusoid. In a parallel fashion, periportal (Zone 1) hepatocytes, regulated primarily by glucagon, have a lower propensity for glycogen storage since glucagon stimulates gluconeogenesis and glycogenolysis (breakdown of glycogen) to release glucose into the bloodstream^37^. In contrast, perivenous (Zone 3) hepatocytes, regulated primarily by insulin, are more adept at storing glycogen since insulin stimulates glycogenesis, the conversion of glucose into glycogen^37^. Here, we sought to replicate such behavior by creating a gradient of insulin/glucagon balance across our microfluidic chamber to pattern hepatocytes to display a corresponding gradient in carbohydrate/glucose metabolism.

**Figure 4:**
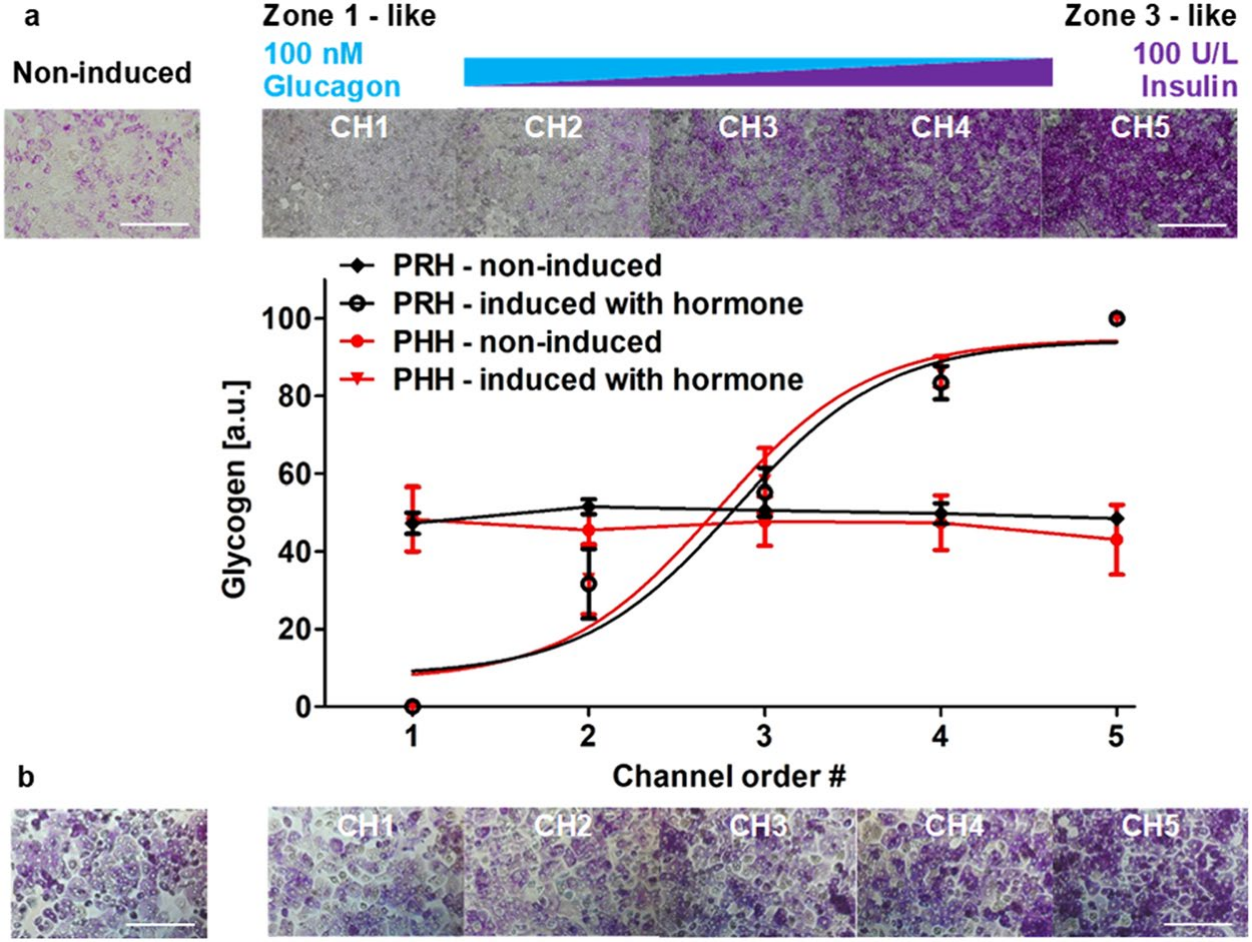
Zonation of primary (**a**) rat and (**b**) human hepatocytes in carbohydrate metabolism in the MPOC platform. Hepatocytes induced with a hormonal gradient (one inlet: 100 nM glucagon, 0 U/L insulin and the other inlet: 0 nM glucagon, 100 U/L insulin) at a flow rate of 120 µL/hr produced a zonation of glycogen across the width of the device. Image quantification for PAS staining of glycogen was analyzed by using Image J. (**a**) All data met p < 0.05 except that non-induced vs ch2, non-induced vs ch3, and ch4 vs ch5 are not significant (ANOVA n = 4, Tukey’s test). (**b**) All data met p < 0.05 except that non-induced vs ch2, non-induced vs ch3, ch2 vs ch3, and ch4 vs ch5 are not significant (ANOVA n = 4, Tukey’s test). Each experiment was replicated using at least three different devices. Scale bar: 400 µm.

Primary rat/human hepatocytes plated in the microfluidic device made a confluent monolayer with a stabilized polygonal morphology after 24 hours (Fig. 2e,f). After inducing the cells in the device with a gradient of 100 nM glucagon and 100 U/L insulin for 24 hours, they were stained with PAS staining reagent. A gradual patterning of glycogen storage in both primary rat and human hepatocytes was observed along the gradient of the insulin-to-glucagon ratio across the width of the device (Fig. 4). While hepatocytes in channel 1 (~100 nM glucagon, 0 U/L insulin) displayed a low intensity in PAS staining thus low glycogen storage, hepatocytes in channel 5 (~0 nM glucagon, 100 U/L insulin) displayed a high intensity thus high glycogen storage. Moderate intensities were observed in the middle channel similar to that of non-induced rat/human hepatocytes in a control experiment. Image quantification showed a linear gradient in glycogen storage demonstrating a gradient of carbohydrate metabolism in hepatocytes (Fig. 4). This microfluidic tissue patterning result, using opposing gradients in insulin and glucagon, is similar to the *in vivo* gradients in carbohydrate/glucose metabolism in the liver sinusoid.

### Zonation of nitrogen metabolism in hepatocytes

**Figure 5:**
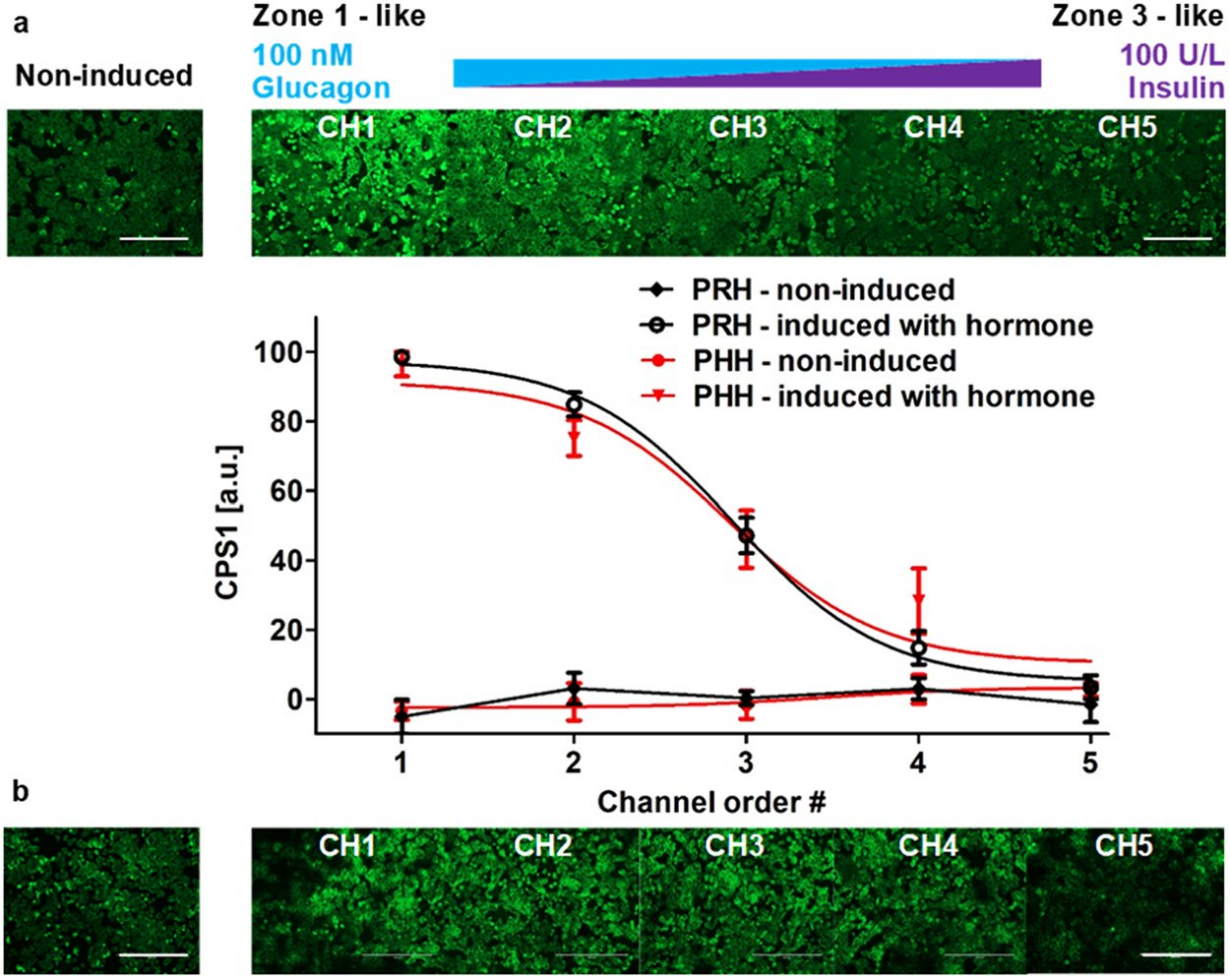
Zonation of primary (**a**) rat and (**b**) human hepatocytes in nitrogen metabolism in the MPOC platform. Hepatocytes induced with a hormonal gradient (one inlet: 100 nM glucagon, 0 U/L insulin and the other inlet: 0 nM glucagon, 100 U/L insulin) at a flow rate of 120 µL/hr displayed a descending CPS1 expression level along with the width of the device. Image quantification of CPS1 staining in hepatocytes induced by a gradient of the insulin-to-glucagon ratio indicated a descending profile of CPS1 expression level which demonstrates nitrogen metabolism. (**a**) All data met p < 0.05 except that non-induced vs ch4, non-induced vs ch5, ch1 vs ch2, and ch4 vs ch5 are not significant (ANOVA n = 4, Tukey’s test). (**b**) All data met p < 0.05 except that non-induced vs ch5, ch1 vs ch2, and ch3 vs ch4 are not significant (ANOVA n = 6, Tukey’s test). Each experiment was replicated using at least three different cell pools. Scale bar: 400 µm.

Nitrogen metabolism in the liver, which involves a conversion of glutamine and ammonia to urea through ornithine/urea cycle, mostly occurs in periportal (Zone 1) hepatocytes of the liver sinusoid *in vivo*^39,40^. This zonal behavior is partially driven by the glucagon/insulin gradients along with other dynamic gradients and developmental signaling events. In order to recapitulate this behavior we created a glucagon-insulin gradient in the presence of hepatocytes in our MPOC device and investigated the activity of carbamoyl phosphate synthetase 1 (CPS1) which plays an important role in the ornithine/urea cycle by catalyzing the synthesis of carbamoyl phosphate from ammonia and bicarbonate^39,40^. Primary rat/human hepatocytes cultured in the MPOC device were induced with the glucagon/insulin gradient for 24 hours. After induction, hepatocytes were fixed and stained with CPS1 antibody to measure the expression level of CPS1 across the culture chamber. Zonal patterning of CPS1 was observed across the width of the MPOC device (Fig. 5).

The zonal behavior for nitrogen metabolism in the MPOC device manifested itself as a high-intensity fluorescence for CPS1 expression in channel 1 where hepatocytes were mostly induced by 100 nM glucagon; a low-intensity fluorescence for CPS1 expression in channel 5 where hepatocytes were mostly induced by 100 U/L insulin; and a gradient in CPS1 expression in the channels between these two extremes. A control experiment - where neither insulin nor glucagon was introduced into the culture medium - for rat/human hepatocytes showed a CPS1 expression level similar to that of the hepatocytes induced without glucagon in channel 5. Image analysis of the CPS1 staining shows quantification of the declining gradient profile of CPS1 expression in the MPOC device in parallel to the decreasing glucagon and increasing insulin levels along the axis of the culture chamber (Fig. 5). These results indicate that an *in vivo* like zonal nitrogen metabolism pattern can be induced, *in vitro*, for primary human and rat hepatocytes by enforcing glucagon/insulin gradients in the MPOC device.

### Zonation of xenobiotic metabolism and zonal drug toxicity response in hepatocytes

Cytochrome P450 (CYP450) enzymes in hepatocytes play an important role in xenobiotic metabolism to biotransform many endogenous and exogenous substances^41,42^. Like many of the other metabolic functions of the liver, the xenobiotic metabolism is highly zonated along the liver sinusoid. In normal physiology these Phase I enzymes are highly expressed and are active in the perivenous (Zone 3) hepatocytes. This zonation is thought to be an adaptive mechanism to protect the majority of the liver from a toxic insult from drugs or other exogenous adverse compounds^43^. Namely, the important manifestation of the zonal expression of the CYP450 enzymes is that some drugs, which are converted into toxic intermediate metabolites by the CYP450s, are selectively toxic in the perivenous (Zone 3) regions of the liver sinusoid. A well-known drug displaying this behavior is acetaminophen (paracetamol) whose toxicity upon an overdose is dependent on the CYP450 activity in hepatocytes^44^. CYP450 enzymes such as CYP2E1, CYP1A2, and CYP3A4 are involved in converting acetaminophen to highly reactive and toxic metabolite N-acetyl-p-benzoquinone imine (NAPQI)^44,45^. The NAPQI formation increased by acetaminophen oxidation ultimately leads to drug-induced hepatotoxicity^46^.

**Figure 6:**
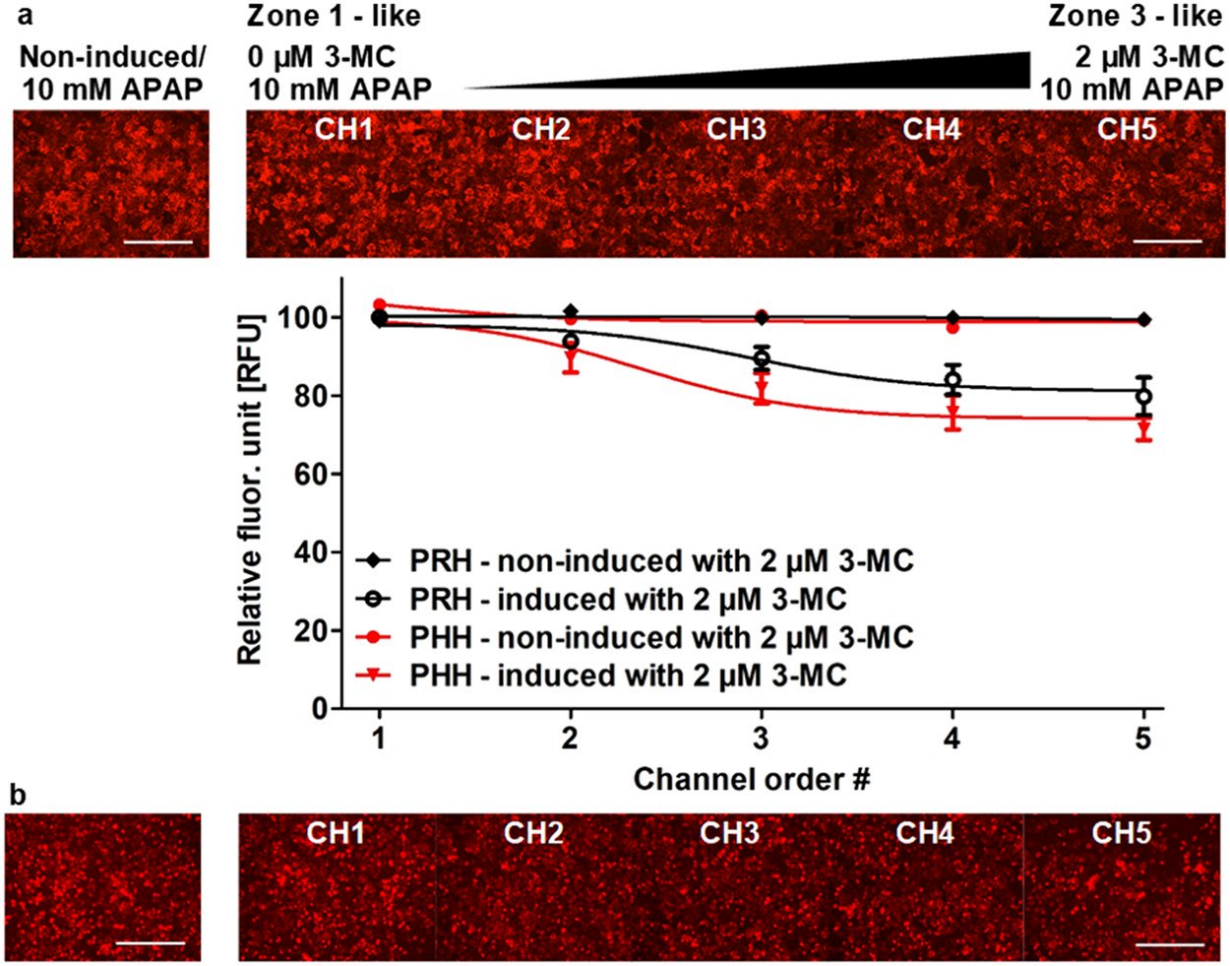
Zonation of primary (**a**) rat and (**b**) human hepatocytes in drug-induced liver toxicity in the MPOC platform. After 24 hours induction with a gradient of 3-MC (one inlet: 0 μM 3-MC and the other inlet: 2 μM 3-MC) at a flow rate of 120 µL/hr, cells were uniformly exposed to a dose of 10 mM acetaminophen (APAP) for 4 hours. Cells were stained with TMRM. The non-induced hepatocytes remained viable while the 3-MC induced hepatocytes relatively lost their viability. The gradient of the acetaminophen-related hepatotoxicity was demonstrated by checking cell viability in CYP activity-dependent response to acetaminophen dose. A gradient of cell viability across the width of the device was estimated through quantification of viability using Image J. (**a**) All data met p < 0.05 except that non-induced vs ch1, non-induced vs ch2, non-induced vs ch3, ch1 vs ch2, ch1 vs ch3, ch2 vs ch3, ch2 vs ch4, ch3 vs ch4, ch3 vs ch5, and ch4 vs ch5 are not significant (ANOVA n = 5, Tukey’s test). (**b**) All data met p < 0.05 except that non-induced vs ch1, non-induced vs ch2, ch1 vs ch2, ch2 vs ch3, ch3 vs ch4, ch3 vs ch5, and ch4 vs ch5 are not significant (ANOVA n = 4, Tukey’s test). Each experiment was replicated using at least three different cell pools. Scale bar: 400 µm.

Here we sought to replicate the zonal pattern of toxicity observed for acetaminophen in our MPOC device by using a gradient of 3-MC, a well-known inducer of CYP1A enzymes and creating a gradual pattern of CYP1A expression along the MPOC device containing rat/human hepatocytes. We specifically focused on CYP1A2 in our characterization since a) it is involved in acetaminophen metabolism and its conversion to the toxic NAPQI metabolite, and b) it is well conserved and its profile of induction is similar among different species (mouse, rat, dog, monkey, and human)^47^. We have chosen the parameters for 3-MC induction in the MPOC device by conducting supplemental experiments on induction potential (CYP1A2 mRNA fold change) of 3-MC on human hepatocytes in macro well plates (Fig. S3) and based on our previous work^13^. After induction, cells were stained with 500 nM TMRM to check cell viability/mitochondrial activity before dosing with acetaminophen. Cells were then exposed to 10 mM, a sublethal dose, of acetaminophen without flow for 4 hours at 37 °C. After dosing acetaminophen, hepatocytes were again stained with 500 nM TMRM to assess change in cell viability/mitochondria activity. A gradient of cell viability/mitochondrial activity in both primary rat (Fig. 6a) and primary human hepatocytes (Fig. 6b) was observed across the width of the culture chamber of the MPOC device. Specifically, while non-induced hepatocytes (Zone 1 like in channel 1) maintained a high, ~100%, viability along with non-induced control groups, hepatocytes induced with 2 µM 3-MC (Zone-3 like in channel 5) displayed a ~80% viability/mitochondrial activity.

**Figure 7:**
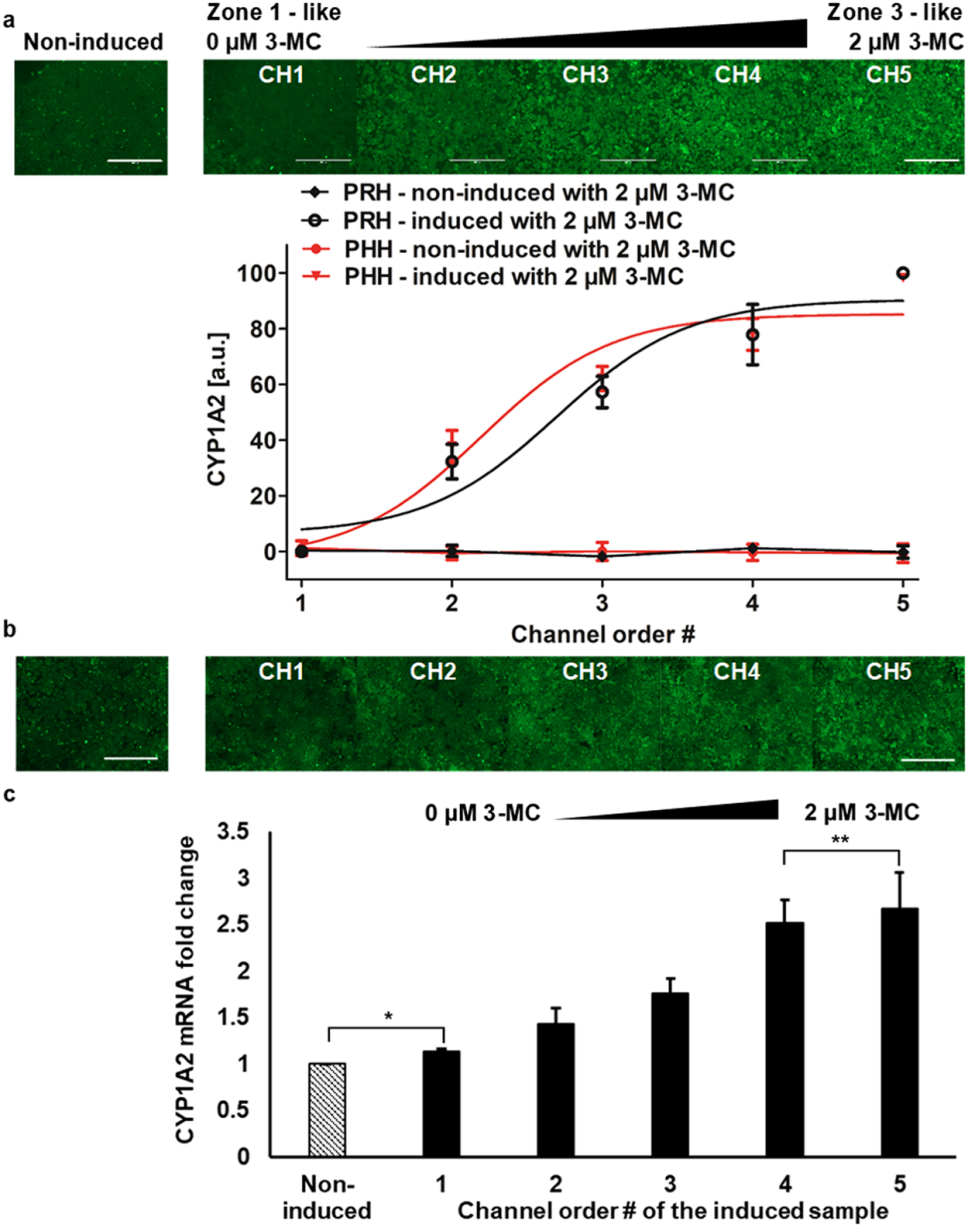
Differential expression of drug metabolic enzymes of primary (**a**) rat and (**b**,**c**) human hepatocytes in the MPOC platform. Hepatocytes cultured in the device were induced with a gradient of 3-MC (one inlet: 0 μM 3-MC and the other inlet: 2 μM 3-MC) at a flow rate of 120 µL/hr for 24 hours. Hepatocytes produced an ascending profile of CYP1A2 expression level along with the width of the device. Image quantification of CYP1A2 staining in hepatocytes was performed by Image J. (**a**) All data met p < 0.05 except that non-induced vs ch1, ch3 vs ch4, and ch4 vs ch5 are not significant (ANOVA n = 3, Tukey’s test). (**b**) All data met p < 0.05 except that non-induced vs ch1 and ch3 vs ch4 are not significant (ANOVA n = 7, Tukey’s test). (**c**) Primary human hepatocytes induced with a gradient of 3-MC produced a zonation of CYP1A2 mRNA fold change along with the width of the device (n = 4, p < 0.05 except for *p = 0.11 and **p = 0.35). Each experiment was replicated using at least three different cell pools. Scale bar: 400 µm.

Upon the demonstration of the zonal toxic response for acetaminophen in the MPOC device, we sought to verify that the observed zonal pattern of toxicity stems from observable differences in CYP1A2 expression. To this end, we conducted two different experiments on hepatocytes to assess the expression of this enzyme along the width of the MPOC chamber following the patterning with 3-MC. First, we used a CYP1A2 antibody staining following a fixation of the primary rat and human hepatocytes with 4% paraformaldehyde (Fig. 7a,b). Next, we conducted a sectional collection of only the human hepatocytes in the MPOC device to assess the mRNA expression of CYP1A2 via RT-qPCR (Fig. 7c). In the control experiments, hepatocytes were cultured under a homogenous level of oxygen, nutrients, and hormones and without any inducer, as in a well plate culture that provides a single zone. Accordingly, the CYP1A2 levels, across the culture chamber are uniform. In the gradient induction experiments, we have observed an ascending profile for the CYP1A2 expression along the width of the MPOC device for both rat and human hepatocytes (Fig. 7a,b) via the antibody staining, and an ascending profile for the human hepatocytes via RT-qPCR (Fig. 7c). As expected this expression profile is countercurrent to the acetaminophen toxicity profile such that higher expression of the CYP1A2 correlates with a lower viability in the Zone-3 like hepatocytes in channel 5. Accordingly, our results indicate that the metabolic patterning approach using the MPOC device can be used to recreate a liver tissue with differentially expressed enzymatic profile along the tissue towards recapitulating a zonal toxicity response for liver cells. While insulin/glucagon ratio can also affect expression and activity of cytochrome enzymes^48^, here, we have not investigated this correlation since our primary objective in analyzing the CYP1A2 expression was to investigate the acetaminophen toxicity in our experiments where we used 3-MC as the inducer. In ongoing work where we aim to look at the effect of different zonal inducers in combination we aim to undertake such analysis.

### Summary and Discussion of Results

In the preceding, we have introduced the idea of microfluidic metabolic patterning in the MPOC device and its use towards recapitulating zonation of several different liver metabolic activities *in vitro*. Through the use of the MPOC device we demonstrated that a) we can generate stable gradients of different metabolic modulators such as hormones and inducers (Figs 4–7) b) we can collect both supernatant and cellular samples in a sectional manner (Figs 2g–i and 7) to assess the variation in metabolic activity and gene expression, an important and necessary feature for spatial characterization c) we can pattern liver tissues with gradually changing activities for carbohydrate/glucose, nitrogen, and xenobiotic metabolisms across the width of the MPOC culture chamber through the use of enforced gradients of either insulin/glucagon ratio or enzymatic inducers. This creation of controlled metabolic patterns using actively enforced concentration gradients in a continuous microfluidic liver tissue model of both rat and human species, along with direct sectional characterization of different metabolic activities constitutes a significant step forward in better understanding heterogeneous tissue response such as the liver zonation we focused on here.

A shortcoming of the current work is the time frame of our experiments where we focus on a 48-hour window to observe metabolic changes both through direct and indirect measures for a variety of different metabolisms. This window is theoretically founded, such that dynamic zonation via inducers and hormones can be achieved in 24–48 hours^49^, and it is practical given the pharmaceutical industry’s focus on short and fast drug screening approaches. Nevertheless, in future studies, we also aim to undertake longer-term studies to create long-term stable metabolic patterns where we further evolve our microfluidic and tissue designs to include multiple chambers^50^ and ultra-thin ECM overlays^51,52^. This approach will then allow us to ask further questions as to the stability and/or disruption of metabolic/zonal patterns in the liver over longer periods and how this affects the overall health of the tissue.

In this work, we have used only one metabolic modulator at a time to investigate the individual effects of each modulator and we limited ourselves to two preliminary cases of these modulators (hormones and enzymatic inducers). Even with this limited approach, our results indicate that the gradual patterns created in our MPOC device recapitulate several aspects of the liver zonation observed *in vivo* in a single connected tissue, similar to the liver sinusoid. Nevertheless, this work can be improved and extended such that we use gradients of multiple modulators at the same time to create a complete zonal response of the liver in a single experiment. Moreover, an addition of other metabolic modulators can be of tremendous use towards capturing zonal variations in additional metabolisms such as lipid metabolism^38^ and further strengthening the patterns in the metabolisms we have already discussed.

Given the recent reports which hail the Wnt/β-catenin - as the “master regulator of metabolic zonation”^53^, and indicate the overwhelming importance of oxygen gradients in the zonation of lipid accumulation, our goal is to add these two classes of modulators into the MPOC framework. Furthermore, while the extreme inputs in our liver MPOC device have provided us with zone-like responses, we believe that by conducting both *in vivo*^38^ and *in silico*^5^ models in parallel to the *in vitro* work, we present here, we can establish a better and direct translation between our work and the *in vivo* zonation phenomena. This, in turn, will result in a high fidelity *in-silico*/*in vitro* liver framework that can negate the need for any further *in vivo* experiments for toxicity and efficacy in line with the goals of the 3 R principles^54^ and the recent push towards using diseases/organs-on-chips^32,55^ for drug screening purposes.

## Conclusions and Outlook

The notion of microfluidic “metabolic patterning” we introduced in this work as well as the MPOC platform we build to achieve it, are versatile and applicable to almost any tissue where gradual or arbitrary patterns in metabolic processes in a continuous tissue are of interest. Here, we have demonstrated the applicability of this metabolic patterning framework by recapitulating some aspects of the liver zonation, the gradual changes in different metabolic functions of liver cells, *in vitro*. However, such gradual patterns of metabolic or other functional activity are not limited to the liver. From the dynamic heterogeneity observed in other mature tissues such as cancer, pancreas, and others^56,57^; to the prominent role of morphogen gradients in developmental biology^2^ towards the creation of such heterogeneity, the notion of gradients and variable patterning in tissues is deeply entrenched in living systems. The current MPOC platform and its future iterations including an expanded number of sections and a 3D tissue structure; and the broad framework of being able to actively enforce metabolic or gene patterns in a tissue can provide a systematic means to study such important physiological phenomena. Since the MPOC platform provides a means to enforce metabolic patterns, it can be re-purposed to study metabolic diseases where one can pattern a tissue with increasing levels of metabolic deficiencies or disruptions. As such, the MPOC platform is not limited to recapitulating *in vivo* phenomena and can also be transformed towards a high-throughput tool for testing different hypotheses on the importance of a particular metabolism or a set of them (i.e. a sensitivity analysis via gradual perturbations).

## Electronic supplementary material

Supplementary document - Metabolic Patterning on a Chip
