# Supplementary material for: Metabolic Patterning on a Chip: Towards in vitro Liver Zonation of Primary Rat and Human Hepatocytes
Source: Sci Rep. 2018 Jun 12;8:8951. doi: 10.1038/s41598-018-27179-6 (PMC5997652; doi:10.1038/s41598-018-27179-6)
Supplement: Supplementary file 1 — Supplementary document - Metabolic Patterning on a Chip [file 41598_2018_27179_MOESM1_ESM.docx]

Metabolic Patterning on a Chip: Towards *in vitro* Liver Zonation of Primary Rat and Human Hepatocytes

*Young Bok (Abraham) Kang^1^, Jinsu Eo^1^,* Safak Mert^1^, *Martin L. Yarmush^1,2^, and O. Berk Usta^1*^*

1. Center for Engineering in Medicine, Department of Surgery, Massachusetts General Hospital, Harvard Medical School, and Shriners Hospitals for Children-Boston, Boston, MA, USA

2. Department of Biomedical Engineering, Rutgers University, 599 Taylor Rd., Piscataway, NJ

08854

**Supplementary documents**


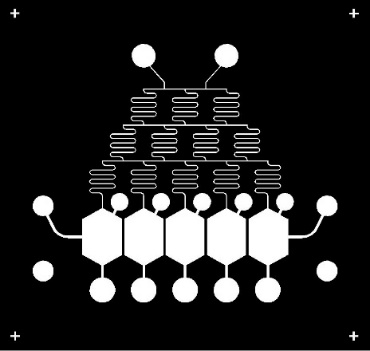


**Fluid inlets**

**Cell inlet**

**Cell outlet**

**Fluid outlets**

**10 mm**

**Barrier**

Supplementary figure 1. Drawing of the MPOC device with barriers.


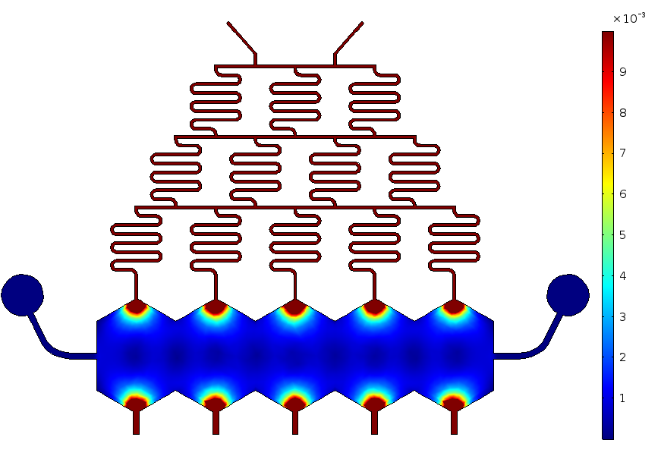


**Ch1**

**Ch2**

**Ch3**

**Ch4**

**Ch5**

Supplementary figure 2. COMSOL^®^ Multiphysics simulation of the flow characteristics in the MPOC device. a), and b) Surface velocity field at a flow rate of 150 µL/hr per each inlet presented at different ranges for clarity. c), and d) Shear stress at a flow rate of 150 µL/hr per each inlet presented at different ranges for clarity. In a), and c) the color map ranges are chosen to clearly show the variation in the Christmas tree section whereas in b), and d) the ranges are adjusted to present variations in the cell culture chamber of the MPOC device.

**Ch1**

**Ch2**

**Ch3**

**Ch4**

**Ch5**

**d)**

**x 10^-3^**

**dyne/cm^2^**

**9**

**8**

**7**

**6**

**5**

**4**

**3**

**2**

**1**

**0**


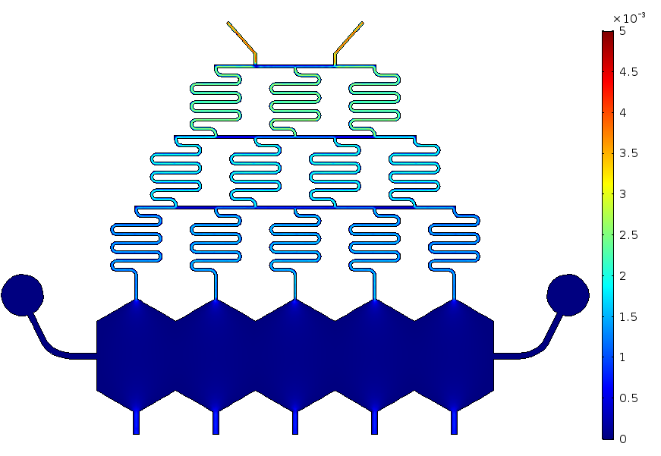

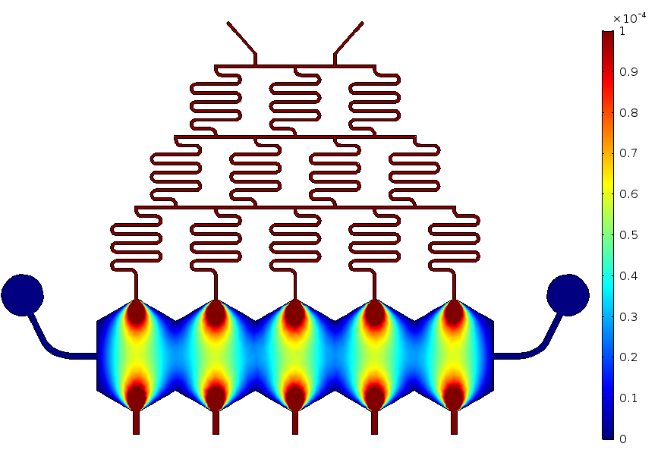

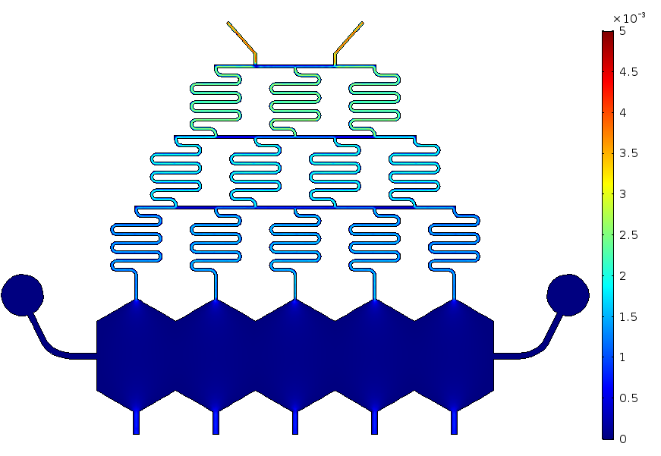


**5**

**4.5**

**4**

**3.5**

**3**

**2.5**

**2**

**1.5**

**1**

**0.5**

**0**

**x 10^-3^ m/s**

**a)**

**1**

**0.9**

**0.8**

**0.7**

**0.6**

**0.5**

**0.4**

**0.3**

**0.2**

**0.1**

**0**

**x 10^-4^ m/s**

**1**

**0.9**

**0.8**

**0.7**

**0.6**

**0.5**

**0.4**

**0.3**

**0.2**

**0.1**

**0**

**dyne/cm^2^**

**Ch1**

**Ch2**

**Ch3**

**Ch4**

**Ch5**

**Ch1**

**Ch2**

**Ch3**

**Ch4**

**Ch5**

**Ch1**

**Ch2**

**Ch3**

**Ch4**

**Ch5**

**b)**

**c)**


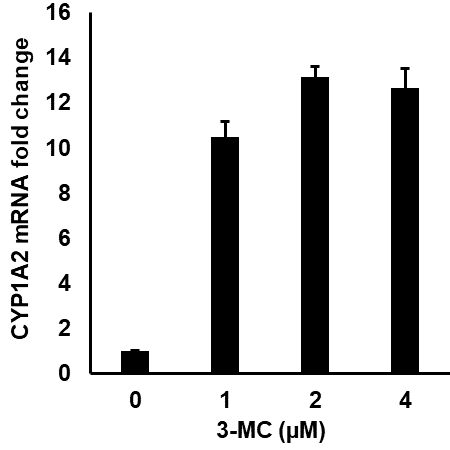


Supplementary figure 3. CYP1A2 mRNA fold change of the 3-MC induced hepatocytes. Primary human hepatocytes cultured in well plates were induced with 3-MC of 0, 1, 2, and 4 µM for 24 hours. The CYP1A2 mRNA fold change of hepatocytes induced with 3-MC of 2 and 4 µM was approximately 13 times higher than the non-induced hepatocytes (ANOVA n=4, p<0.05). Based on these outcomes, hepatocytes in the MPOC device were induced with a gradient of 0-2 µM 3-MC. Each experiment was replicated using at least three different cell pools.
